# Integrated Transcriptomic and Metabolomic Analyses Reveal Key Genes Involved in Phenylpropanoid Metabolism in *Lonicera macranthoides* Flowers

**DOI:** 10.3390/genes16111339

**Published:** 2025-11-06

**Authors:** Zhengchun Li, Zijing Zhou, Hua Feng, Yong Wang, Zhaohua Zeng, Qiandong Hou, Luonan Shen

**Affiliations:** 1 Key Laboratory of Conservation, Development and Utilization of Karst Characteristic Biological Resources, Department of Modern Agriculture, Zunyi Vocational and Technical College, Zunyi 563006, China; zcliah@163.com (Z.L.); fenghua781014@163.com (H.F.); wykarst@163.com (Y.W.); zhaohuazeng@163.com (Z.Z.); 2 Institute for Forest Resources & Environment of Guizhou/College of Forestry, Guizhou University, Guiyang 550025, China; zhouzijing15@163.com; 3 School of Ethnic-Minority Medicine, Guizhou Minzu University, Guiyang 550025, China; qiandhou@gzmu.edu.cn

**Keywords:** *Lonicera macranthoides*, transcriptome, metabolome, chlorogenic acids (CGAs), phenylpropanoid biosynthesis

## Abstract

Background: *Lonicera macranthoides* (*L. macranthoides*) is a traditional Chinese medicinal plant, the flower buds of which are rich in bioactive compounds, such as chlorogenic acids (CGAs) and flavonoids, and exhibit diverse pharmacological activities. Methods: Integrated transcriptomic and metabolomic analyses were conducted across three floral developmental stages: flower bud (FB), white flower (WF), and golden flower (GF). Results: Clustering analysis revealed distinct molecular profiles, with the WF and GF stages clustering together and clearly separating from the FB stage. The most significant metabolomic variation was observed between the GF and FB stages. KEGG enrichment analysis consistently highlighted the phenylpropanoid biosynthesis pathway as a key hub. Stage-specific accumulation patterns were identified for critical metabolites: caffeoylquinic acid peaked at the WF stage, while ferulic acid, sinapic acid, cinnamic acid, and p-coumaric acid reached their highest levels at the GF stage. Within this pathway, we identified 11 DEGs encoding the core enzymes, alongside 15 associated DAMs. The expression trends of four *PAL* genes were fully consistent with the accumulation of related precursors, and *F5H* expression correlated with its downstream product, sinapic acid. WGCNA identified a key module highly correlated with key phenolic acids, containing 71 transcription factors, including MYB, bHLH, WRKY, and AP2/ERF families, potentially forming a complex regulatory network for phenylpropanoid biosynthesis. Conclusions: This study deciphers the stage-specific regulatory network of CGA biosynthesis, providing critical insights and genetic resources for cultivating high-medicinal-content varieties of *L. macranthoides*.

## 1. Introduction

*Lonicera macranthoides* Hand.-Mazz. (*L. macranthoides*), a traditional medicinal plant indigenous to China, is extensively distributed throughout the southern regions of the country [1,2]. This plant demonstrates a range of pharmacological activities, including antibacterial, antioxidant, antipyretic, hepatoprotective, and antitumor effects, while maintaining low toxicity. Its medicinal efficacy is mainly attributed to the active secondary metabolites it contains, typically chlorogenic acid (CGA)-related compounds, flavonoids, and alkaloids [1,3,4,5]. In addition, the flowers of *L. macranthoides* are the principal medicinal component for clinical application [6]. Although *L. macranthoides* and *Lonicera japonica* Thunb belong to the same *Lonicera* family, there are significant differences in their chlorogenic acid contents. Studies have shown that the content of chlorogenic acid in the flowers of *L. macranthoides* is significantly higher than that in *L. japonica*, with a difference of 1.7 to 2 times [7].

The biosynthesis of CGAs is mainly accomplished through the phenylpropanoid metabolic pathway, a key branch of plant secondary metabolism [8,9]. Research indicates that the biosynthesis of chlorogenic acid in *L. macranthoides* begins with phenylalanine (Phe), which undergoes a series of enzymatic reactions to ultimately form CGAs. Initially, phenylalanine is deaminated to cinnamic acid via phenylalanine ammonia-lyase (PAL) [10,11]. Cinnamic acid is then hydroxylated to p-coumaric acid via cinnamate-4-hydroxylase (C4H). p-Coumaric acid is activated to p-coumaroyl-CoA via 4-coumarate-CoA ligase (4CL), and this product is further hydroxylated to caffeoyl-CoA via p-coumarate-3-hydroxylase (C3H) [12,13,14]. Finally, caffeoyl-CoA undergoes an acylation reaction with quinic acid, catalyzed via hydroxycinnamoyl-CoA quinate transferase (HQT), resulting in the production of chlorogenic acid (3-O-caffeoylquinic acid) [15,16].

However, the specific regulatory mechanisms governing CGA biosynthesis in plants remain poorly characterized. Moreover, in practical applications, the arbitrary selection of herb harvesting times, due to a lack of precise understanding of CGAs accumulation patterns, poses challenges for the quality control of *L. macranthoides* medicinal materials. The rapid advancement of multi-omics technologies offers a novel research approach for elucidating the accumulation patterns of secondary metabolites in medicinal plants and addressing these challenges.

To further reveal regulating mechanisms underlying CGA biosynthesis and provide candidate genes for cultivating high medicinal-content varieties of *L. macranthoides*, this study conducted integrated transcriptome and metabolome analyses targeting three key floral developmental stages: flower bud (FB), white flower (WF), and golden flower (GF). It focused on analyzing core differentially expressed genes (DEGs) and differentially accumulated metabolites (DAMs) in the phenylpropanoid biosynthesis pathway. Additionally, weighted gene co-expression network analysis (WGCNA) was used to screen candidate transcription factors that potentially regulate CGA biosynthesis.

## 2. Materials and Methods

### 2.1. Plant Materials

**Figure 1:**
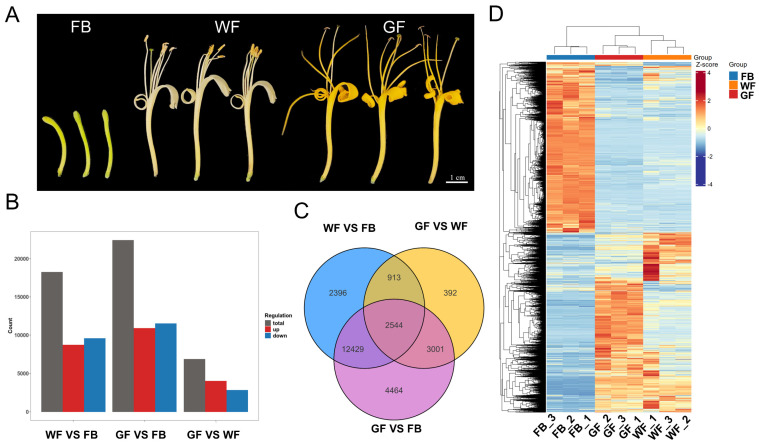
Analysis of differentially expressed genes (DEGs) in the transcriptome of *L. macranthoides* at different floral developmental stages: (**A**) Three floral developmental stages of *L. macranthoides*: flower bud (FB), white flower (WF), and golden flower (GF). (**B**) The number of upregulated and downregulated DEGs in different comparison groups. (**C**) A Venn diagram of the DEGs among the different groups. (**D**) Clustering heatmap of gene expression across three floral developmental stages. In group comparisons, the group before “vs.” is the comparison group, and the group after “vs.” is the control group.

The experimental materials in this study were collected from the *Lonicera macranthoides* Germplasm Resource Nursery in Zhengchang Town, Suiyang County, Zunyi City, Guizhou Province, China (27.89° N, 107.25° E; elevation 826 m; plant spacing of 2 m and row spacing of 2.5 m), during June to July 2025. The collected samples were from three key floral developmental stages of *L. macranthoides* (Figure 1A), specifically: flower bud (FB), white flower (WF), and golden flower (GF). All samples were quickly frozen in liquid nitrogen immediately after collection and then transferred to a −80 °C refrigerator for long-term storage for subsequent RNA extraction and metabolite detection.

### 2.2. RNA Extraction and Quality Control

Total RNA was extracted using a CTAB-PBIOZOL reagent combined with the ethanol precipitation method [17]. After extraction, the concentration of total RNA was determined using a Qubit Fluorometer (Thermo Fisher Scientific, Waltham, MA, USA), and RNA integrity was evaluated with a Qsep400 High-Throughput Bio-Fragment Analyzer (BiOptic, New Taipei City, Taiwan) to ensure an RNA Integrity Number (RIN) > 8.0. Only high-quality RNA samples were used for subsequent experiments.

### 2.3. cDNA Library Construction and Sequencing

cDNA libraries were constructed using qualified total RNA as templates. The library construction process was as follows: first, cDNA was synthesized via reverse transcription (Takara PrimeScript™ Reverse Transcription Kit, Kusatsu, Japan); then, isothermal amplification was performed using phi29 DNA polymerase to generate DNA nanoballs (DNBs) with more than 300 copies per molecule. The prepared DNBs were uniformly loaded onto a flow cell and sequenced on the BGI DNBSEQ-T7 sequencing platform with paired-end reads.

### 2.4. Read Processing and De Novo Transcriptome Assembly

Raw sequencing reads were quality-filtered using fastp software (v0.23.1) [18] based on the following criteria: Reads containing adapter sequences were removed. Paired-end reads were discarded if the proportion of N bases in either read exceeded 10% of the total bases in that read. Paired-end reads were discarded if the proportion of low-quality bases (Q ≤ 20) in either read exceeded 50% of the total bases in that read. All subsequent analyses were performed using high-quality clean reads. De novo transcript assembly of clean reads was conducted with Trinity software (v2.15.1) (https://github.com/trinityrnaseq/trinityrnaseq, accessed on 11 August 2025) [19], and the assembled transcripts were clustered and deduplicated using Corset software (v1.09) [20] to obtain a set of non-redundant transcripts. Open reading frames (ORFs) and coding sequences (CDS) of non-redundant transcripts were predicted using TransDecoder software (v5.5.0) (https://github.com/TransDecoder/, accessed on 11 August 2025), and the corresponding amino acid sequences were obtained simultaneously [21].

### 2.5. Functional Annotation of Genes

Functional Annotation: Non-redundant transcript sequences were aligned against six databases—Kyoto Encyclopedia of Genes and Genomes (KEGG) (https://www.genome.jp/kegg/, accessed on 15 August 2025) [22], non-redundant protein (NR) (http://www.ncbi.nlm.nih.gov, accessed on 17 August 2025) [23], Swiss-Prot protein (http://www.expasy.ch/sprot, accessed on 20 August 2025), Gene Ontology (GO) (http://www.geneontology.org/, accessed on 22 August 2025) [24], euKaryotic Orthologous Groups (KOG) (https://www.ncbi.nlm.nih.gov/research/cog/, accessed on 25 August 2025) [25], and Translation of EMBL nucleotide sequence database (TrEMBL) (https://www.uniprot.org/, accessed on 27 August 2025) [26]—using DIAMOND software (v2.1.6). An e-value cutoff of 1 × 10^−5^ was applied to filter alignment results; this threshold is widely accepted in homologous sequence identification studies, as it effectively minimizes false-positive matches (i.e., reducing the probability of random sequence similarity) while ensuring the retention of biologically meaningful homologous sequences [27]. Predicted amino acid sequences were matched against the Pfam database (https://pfam.xfam.org/, accessed on 29 August 2025) using HMMER software (v3.3.2) for domain identification [28,29]. Comprehensive functional annotations of transcripts from seven databases were integrated.

### 2.6. Expression Quantification and Differential Expression Analysis

Expression Quantification: Read counts for each transcript were calculated using RSEM software (v1.3.3), and the Fragments Per Kilobase of transcript per Million mapped reads (FPKM) was computed based on transcript length to quantify transcript expression levels [30,31]. Differential Expression Analysis: Differential expression analysis between pairwise sample groups (WF vs. FB, GF vs. FB, and GF vs. WF) was performed using DESeq2 software (v1.38.3). The Benjamini–Hochberg method was used to adjust *p*-values (adjusted *p*-value, padj) for multiple testing. Transcripts with padj < 0.05 and |log_2_ (fold change)|> 1 were defined as differentially expressed genes (DEGs). Enrichment Analysis: GO functional enrichment and KEGG pathway enrichment analyses were conducted based on the hypergeometric test. GO enrichment was performed at the GO term level, and KEGG enrichment was performed at the pathway level. Terms or pathways with padj < 0.05 were considered significantly enriched.

### 2.7. Sample Preparation and UPLC-MS/MS Analysis

*L. macranthoides* floral samples stored at −80 °C were transferred to a freeze dryer (Scientz-100F, Ningbo Xinzhi, Ningbo, China) for vacuum freeze-drying. The dried samples were ground into a homogeneous powder using a grinder (MM 400, Retsch, Haan, Germany). Exactly 50 mg of sample powder was weighed, and 1200 μL of pre-chilled (−20 °C) 70% methanol–water internal standard extraction solution (containing internal standards to correct extraction efficiency) was added. The supernatant was aspirated and filtered through a 0.22 μm pore size microfiltration membrane (organic phase, Millipore, Burlington, MA, USA), and the filtrate was collected into a sample vial for subsequent UPLC-MS/MS analysis. An ultra-high performance liquid chromatography–tandem mass spectrometry (UPLC-MS/MS) system (LC-30A, Shimadzu, Kyoto, Japan; equipped with a triple quadrupole mass spectrometer detector) was used for metabolite separation and detection.

### 2.8. Data Preprocessing and Quality Control

The XCMS program (R package, v3.18.0) was used for peak extraction, peak alignment, and retention time correction of raw mass spectrometry data. Metabolic peaks with a missing rate > 50% across all samples were filtered out (to avoid interference from low-reliability data). A hierarchical imputation strategy was applied to missing values of remaining peaks: peaks with a missing rate > 50% were imputed with “1/5 of the minimum value in the group”, and peaks with a missing rate ≤ 50% were imputed using the K-nearest neighbor (KNN) algorithm. The filled peak areas were corrected using the support vector regression (SVR) method to eliminate errors caused by instrument drift.

### 2.9. Metabolite Identification and Dataset Construction

Corrected peaks were matched and identified by (1) searching the laboratory’s in-house standard metabolite database (matching retention time, accurate mass-to-charge ratio, and MS/MS fragment ions); (2) integrating public databases (Metlin, HMDB, MassBank); (3) conducting structural prediction using the metDNA metabolite annotation tool. Finally, metabolites with a comprehensive identification score ≥ 0.5 and a coefficient of variation (CV) < 0.5 in QC samples were selected (to ensure identification reliability and reproducibility). For the same metabolite detected in both positive and negative ion modes, the entry with the “highest qualitative grade and smallest CV in QC samples” was retained to form the final metabolite dataset.

### 2.10. Statistical Analysis and Differential Metabolite Screening

PCA was performed on normalized metabolite data using SIMCA-P 14.1 software (Umetrics, Umeå, Sweden). Score plots were used to evaluate the overall metabolic differences between groups and the degree of variation within groups, verifying sample reproducibility (samples with high intra-group aggregation and clear inter-group separation were considered qualified). Orthogonal Partial Least Squares–Discriminant Analysis (OPLS-DA) was used to calculate the Variable Importance in Projection (VIP) values, with VIP ≥ 1 set as the threshold—this is a widely accepted criterion in multivariate analysis, as it effectively identifies variables that contribute significantly to group discrimination. Differentially accumulated metabolites (DAMs) were screened by combining VIP values with fold change: fold change ≥2 or ≤0.5 was adopted to ensure the selected metabolites had biologically meaningful concentration differences, while avoiding false positives from minor fluctuations, consistent with standard practices in metabolomics studies.

### 2.11. Weighted Gene Co-Expression Network Analysis

The “WGCNA Shiny Analysis Plugin” in TBtools-II software was used [32]. Metabolite relative contents and transcriptome gene expression levels (FPKM values) were used as input data to construct a weighted gene co-expression network, and modules and candidate regulatory genes significantly correlated with chlorogenic acid (CGA)-related metabolite contents were screened.

## 3. Results

### 3.1. Transcriptome Analysis of Floral Developmental Stages

To investigate gene expression changes in *L. macranthoides* across three floral developmental stages, transcriptome analysis was conducted on samples from the flower bud (FB), white flower (WF), and golden flower (GF) stages (Figure 1A). After removing low-quality reads, a total of 584,576,466 clean reads were obtained, resulting in 99,712 unigenes. The Q30 base percentage ranged from 97.01% to 97.42%, and the GC content ranged from 42.83% to 45.33%, indicating the high quality of the transcriptome sequencing data (Table S1).

To obtain comprehensive gene function annotations, a total of 99,712 genes were analyzed against the Nr, Nt, Pfam, KOG, Swiss-Prot, KEGG, and GO databases. The results showed that 66,922 genes were annotated in at least one database, and all annotation results are provided in Table S2. Among these, 63,321 genes (63.50%) were annotated via the Nr database. Sequence homology alignment revealed that 13,216 sequences (20.87%) showed significant matches to *Nyssa sinensis*, 4641 sequences (7.33%) matched *Camellia sinensis*, followed by *Camellia lanceoleosa* (3558 sequences, 5.62%), *Actinidia chinensis* var. chinensis (2718 sequences, 4.29%), and *Vitis vinifera* (2270 sequences, 3.58%); additionally, 44.59% of the sequences were homologous to those from other species (Figure S1). A total of 56,034 genes (56.20%) were annotated in the GO database. GO enrichment analysis indicated that the genes were mainly enriched in terms such as cellular process, metabolic process, response to stimulus, cellular anatomical entity, protein-containing complex, binding, and catalytic activity under the Biological Process (BP), Molecular Function (MF), and Cellular Component (CC) categories (Figure S2). For the KEGG database, 50,898 genes (51.05%) were annotated, and the top three KEGG pathways were general function prediction only (9211 genes), signal transduction mechanisms (4709 genes), and posttranslational modification, protein turnover, chaperone (4238 genes) (Figure S3).

Pairwise comparisons of the transcriptome data among the different floral developmental stages were performed using the criteria for differentially expressed genes (DEGs): |fold change| > 2 and corrected *p*-value < 0.05. A total of 18,282 DEGs (8702 upregulated and 9580 downregulated) were identified in the WF vs. FB comparison, 22,438 DEGs (10,898 upregulated and 11,540 downregulated) in the GF vs. FB comparison, and 6850 DEGs (4030 upregulated and 2820 downregulated) in the GF vs. WF comparison (Figure 1B). A Venn diagram was used to classify the identified DEGs, revealing that 2544 DEGs were shared among the three stages (Figure 1C). A clustering analysis heatmap provided an overview of expression patterns exhibited by DEGs across the three floral developmental stages of *L. macranthoides*. As shown in Figure 1D, the three stages could be divided into two clusters based on gene expression levels: WF and GF were grouped into one cluster, while FB formed a separate cluster due to significant differences in gene expression compared to the other two stages. These results demonstrated significant differences in the transcriptional level of different floral developmental stages in *L. macranthoides*.

### 3.2. Functional Enrichment Analysis of DEGs at Three Floral Developmental Stages

**Figure 2:**
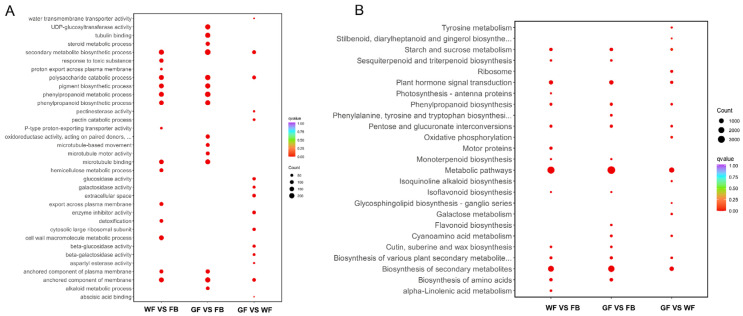
The GO and KEGG enrichment analyses of DEGs at different floral developmental stages of *L. macranthoides*: (**A**) GO enrichment plots of DEGs. (**B**) KEGG enrichment plots of DEGs. FB: flower bud; WF: white flower bud; GF: golden flower bud. In group comparisons, the group before “vs.” is the comparison group, and the group after “vs.” is the control group.

For a deeper exploration of the biological functions of DEGs across the three floral developmental stages, enrichment analysis of these genes was carried out with the Gene Ontology (GO) and Kyoto Encyclopedia of Genes and Genomes (KEGG) databases (Figure 2A,B). The analysis revealed that GO functional terms were mainly enriched in the anchored component of the membrane, phenylpropanoid biosynthetic process, and secondary metabolite biosynthetic process, and KEGG pathways were mainly enriched in biosynthesis of secondary metabolites, plant hormone signal transduction, and phenylpropanoid biosynthesis. Notably, secondary metabolite biosynthetic process and phenylpropanoid biosynthesis were identified in both GO and KEGG enrichment analyses. These results suggest that the expression of genes related to secondary metabolite biosynthesis differs significantly among the three floral developmental stages, indicating differences in secondary metabolite composition at different developmental stages of *L. macranthoides*.

### 3.3. Metabolome Analysis of Three Floral Developmental Stages

**Figure 3:**
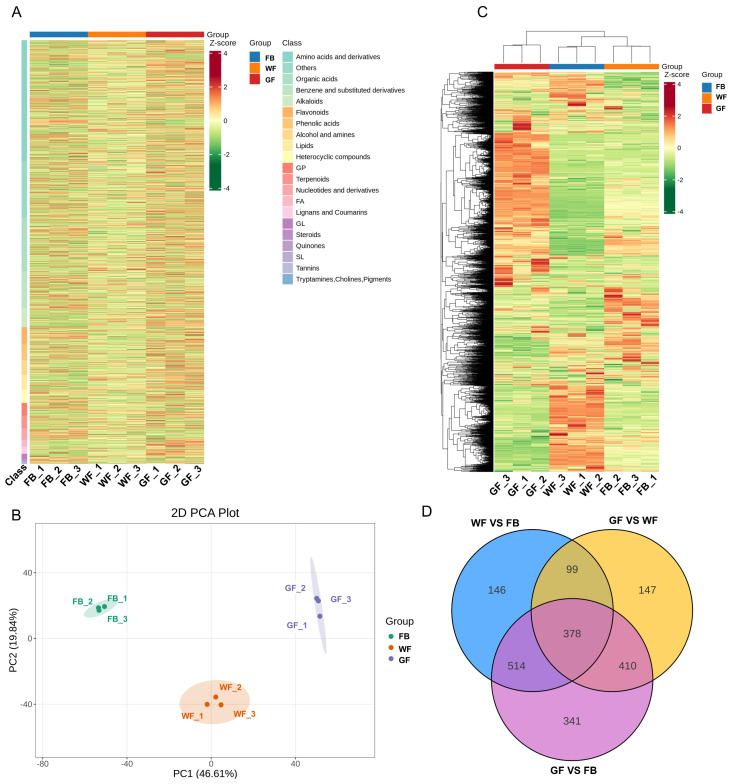
Analysis of differentially accumulated metabolites (DAMs) at different floral developmental stages of *L. macranthoides*: (**A**) The overall clustering plot of metabolome samples. (**B**) PCA plot of metabolome samples. PC1 represents the first principal component. PC2 represents the second principal component. The percentage indicates the explanatory rate of the principal component to the dataset; each point in the plot represents one sample. Samples from the same group are labeled with the same color. Group denotes the grouping. (**C**) Clustering heatmap of DAMs. (**D**) Venn diagram of DAMs among different comparison groups. In group comparisons, the group before “vs.” is the comparison group, and the group after “vs.” is the control group.

To further analyze the differences in metabolites of *L. macranthoides* at three floral developmental stages, an untargeted metabolomics analysis was conducted using an LC-ESI-MS/MS system, leading to the detection of 4304 metabolites (Figure 3A). These metabolites were classified into 21 classes, specifically including 1227 amino acids and derivatives; 372 benzene and substituted derivatives; 153 alcohols and amines; 157 phenolic acids; 129 glycerophospholipids; 46 glycerolipids; 116 nucleotides and derivatives; 175 flavonoids; 10 quinones; 68 lignans and coumarins; 9 sphingolipids; 8 tannins; 4 tryptamines, cholines, and pigments; 195 alkaloids; 129 terpenoids; 553 organic acids; 138 heterocyclic compounds; 24 steroids; 74 fatty acyls; and 146 lipids.

Principal component analysis (PCA) revealed that the metabolome samples from the three floral developmental stages could be distinctly separated into three groups (Figure 3B), suggesting that different floral developmental stages significantly influence metabolite accumulation. Differentially accumulated metabolites (DAMs) were identified among the three sample comparisons (WF vs. FB, GF vs. FB, and GF vs. WF) using the screening criteria of variable importance in projection (VIP) ≥ 1 and fold change ≥ 2 or fold change ≤ 0.5. A total of 1137 differential DAMs were found (Table S2) in the WF vs. FB comparison (378 upregulated, 759 downregulated); 1643 differential DAMs in the GF vs. FB comparison (588 upregulated, 1055 downregulated); and 1034 differential DAMs in the GF vs. WF comparison (342 upregulated, 692 downregulated).

The clustering analysis heatmap (Figure 3C) provided an overall perspective on the expression patterns of DAMs at the three floral developmental stages of *L. macranthoides*. The results showed that these DAMs could be clustered into two groups: WF and FB were grouped together, while GF formed a separate group due to obvious differences from the other two stages. As illustrated in the Venn diagram (Figure 3D), 378 DAMs were shared among the three floral developmental stages, demonstrating significant changes in metabolite accumulation at different floral developmental stages.

### 3.4. Integrated Analysis of Genes and Metabolites in the Phenylpropanoid Biosynthesis Pathway

**Figure 4:**
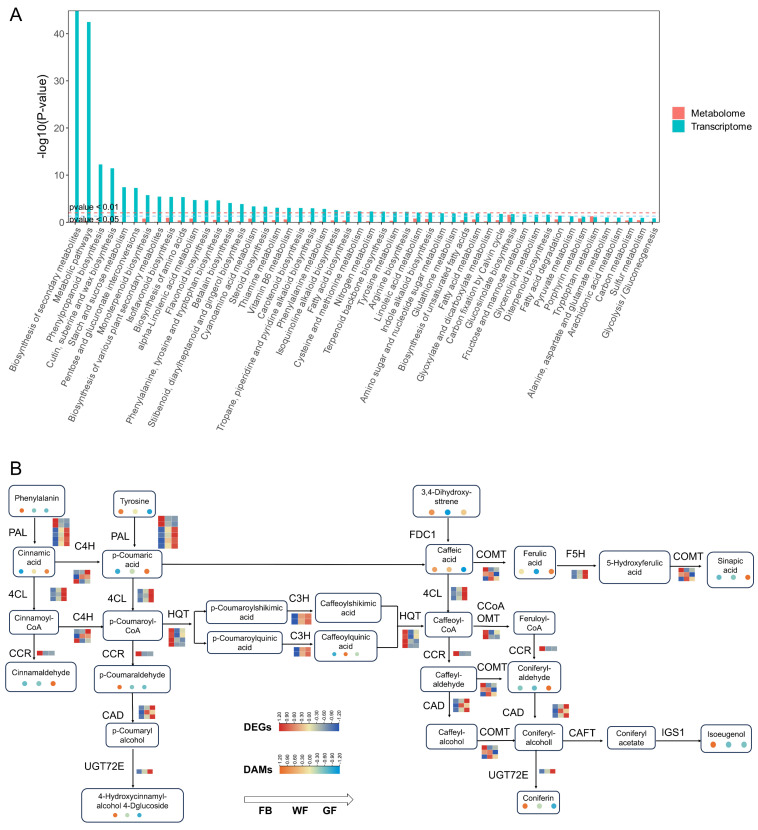
Integrated analysis of DEGs and DAMs in the phenylpropanoid biosynthesis pathway: (**A**) Bar charts of KEGG enrichment analysis for co-enriched KEGG pathways from transcriptome and metabolome. (**B**) Heatmaps of DEGs and DAMs in the phenylpropanoid biosynthesis pathway.

In this study, bar charts were generated to illustrate the co-enrichment of KEGG pathways by integrating transcriptome and metabolome data (Figure 4A). These charts depict the enrichment *p*-values for each pathway. The findings revealed that biosynthesis of secondary metabolites, metabolic pathways, and phenylpropanoid biosynthesis were co-enriched pathways in both omics datasets. To further investigate the variation patterns of genes and metabolites in the phenylpropanoid biosynthesis pathway of *L. macranthoides* at three floral developmental stages, this study integrated the associations among transcriptome data, metabolome data, and metabolic pathways, analyzed the interaction between DEGs and DAMs, and thereby inferred the potential mechanism of action between metabolites and genes (Figure 4B). Based on the phenylpropanoid biosynthesis pathway, 15 differentially accumulated metabolites (DAMs) were identified, including tyrosine, phenylalanine, caffeoylquinic acid, ferulic acid, sinapic acid, p-coumaraldehyde, cinnamaldehyde, 3,4-dihydroxystyrene, isoeugenol, coniferin, cinnamic acid, p-coumaric acid, caffeic acid, 4-hydroxycinnamyl alcohol 4-D-glucoside, and coniferyl aldehyde. Additionally, 11 DEGs were screened, namely phenylalanine ammonia-lyase (*PAL*), 4-coumarate-CoA ligase (*4CL*), cinnamoyl-CoA reductase *(CCR)*, cinnamate-4-hydroxylase (*C4H*), caffeic acid O-methyltransferase *(COMT)*, ferulate 5-hydroxylase *(F5H)*, hydroxycinnamoyl-CoA quinate transferase (*HQT*), p-coumarate 3-hydroxylase (*C3H*), cinnamyl alcohol dehydrogenase *(CAD)*, UDP-glycosyltransferase 72E *(UGT72E)*, and caffeoyl-CoA O-methyltransferase *(CCoAOMT)*.

**Figure 5:**
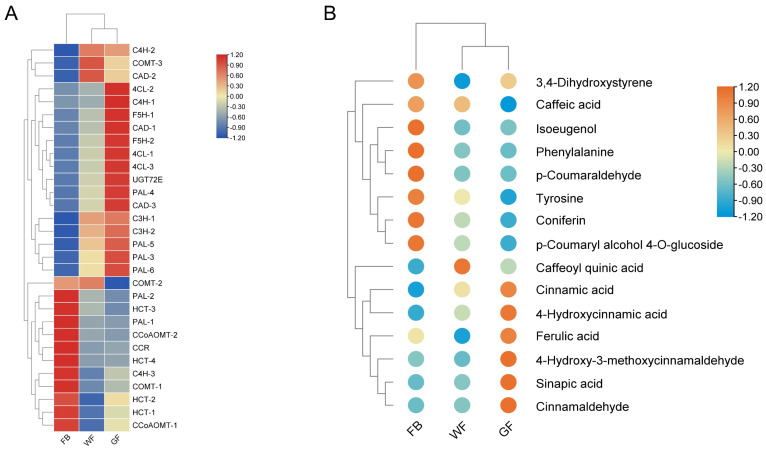
Clustering heatmap of DAMs and DEGs: (**A**) Clustering heatmap of DEGs in the phenylpropanoid biosynthesis pathway. (**B**) Clustering heatmap of DAMs in the phenylpropanoid biosynthesis pathway.

Clustering analysis results showed that both DAMs and DEGs could be divided into two clusters (Figure 5A,B): the white flower (WF) and golden flower (GF) stages were grouped into one cluster, while the flower bud (FB) stage formed a separate cluster. Among chlorogenic acid-related metabolites, all were clustered into one group, except caffeic acid. Within this group, the relative content of caffeoylquinic acid was higher at the WF stage, whereas the other four metabolites (ferulic acid, sinapic acid, cinnamic acid, and p-coumaric acid) reached their highest relative contents at the GF stage. For the *PAL* gene family, the expression levels of four members (*PAL3-PAL6*) exhibited a consistent increasing trend corresponding to the content changes in downstream metabolites (cinnamic acid and p-coumaric acid), while showing an opposite trend to the content changes of upstream metabolites (phenylalanine and tyrosine). The expression level of *F5H* also demonstrated a consistent increasing trend with its product (sinapic acid). p-Coumaroyl-CoA is catalyzed via HCT to form p-coumaroyl quinic acid, which is further catalyzed via C3H to generate caffeoyl quinic acid. Meanwhile, HCT can also catalyze caffeoyl quinic acid to produce caffeoyl-CoA. The results show that HCT1 and HCT2 exhibit an opposite trend to the relative content of caffeoyl quinic acid metabolites; thus, it is inferred that their function is to catalyze the conversion of caffeoyl quinic acid to caffeoyl-CoA. HCT is highly expressed during the FB stage, while the expression level of C3H is very low—this may account for the relatively low relative content of caffeoyl quinic acid in the FB stage. Subsequently, as the expression of WF-related genes increases, its product (i.e., caffeoyl quinic acid) begins to accumulate, and its content rises. Later, during the GF stage, C3H maintains high expression, and the expression levels of HCT also increase, leading to a decrease in the relative content of caffeoyl quinic acid. To sum up, under the combined influence of HCT and C3H across the three flower development stages, caffeoyl quinic acid reaches the highest relative content in the WF stage.

### 3.5. Identification of Candidate Transcription Factors Involved in Chlorogenic Acid Biosynthesis

**Figure 6:**
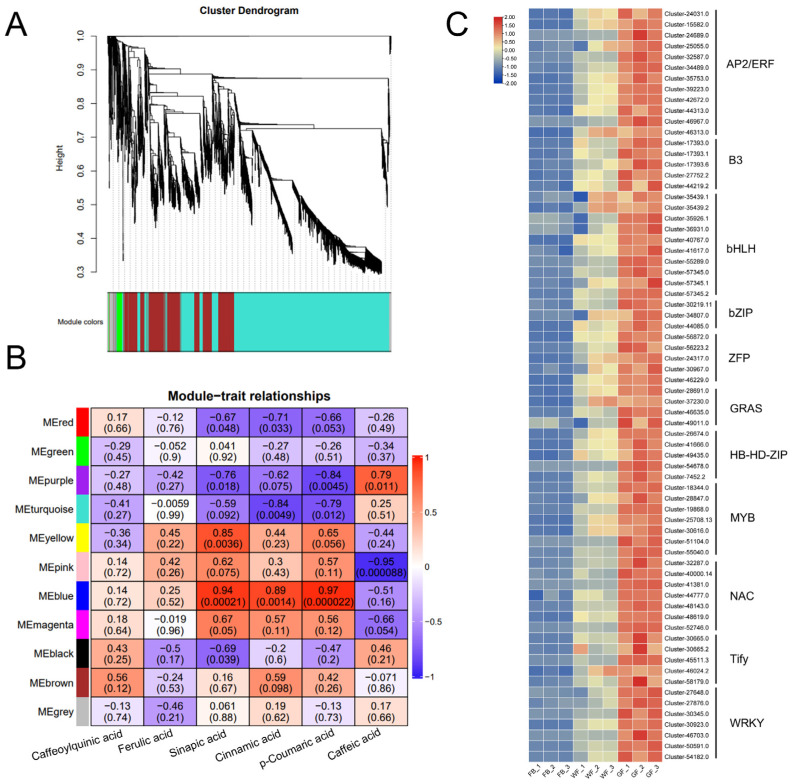
The weighted gene co-expression network analysis (WGCNA) associated with Chlorogenic acid (CGA)-related metabolites in *L. macranthoides*: (**A**) Dendrogram of modules identified via weighted gene WGCNA combined with gene expression clustering dendrogram. (**B**) Module-CGA weight correlations and corresponding *p*-values. The weighted correlations between WGCNA co-expression modules and CGAs and their corresponding significance *p*-value. (**C**) Transcription factor expression heatmap.

In plants, phenylpropanoid metabolism is generally controlled by transcription factors that focus on the structural genes of regulatory enzymes. In this study, a weighted gene co-expression network was constructed with WGCNA (Figure 6A). By integrating the analysis of relative contents of six chlorogenic acid (CGA)-related compounds across three floral developmental stages of *L. macranthoides*, candidate transcription factors regulating CGA biosynthesis were screened out (Figure 6B). Among the co-expression modules, the purple module demonstrated a positive correlation with the relative content of caffeic acid (Pearson correlation coefficient = 0.79), while the blue module exhibited highly positive correlations with the content variations of sinapic acid, p-coumaric acid, and cinnamic acid, with correlation coefficients of 0.94, 0.89, and 0.97, respectively.

Further analysis of genes in the blue module revealed 71 transcription factors, including 12 from the AP2/ERF family, 5 from the B3 family, 10 from the bHLH family, 3 from the bZIP family, 5 from the ZFP family, 4 from the GRAS family, 5 from the HB-HD-ZIP family, 7 from the MYB family, 7 from the NAC family, 5 from the Tify family, and 7 from the WRKY family. The expression heatmap results (Figure 6C) revealed that the expression patterns of these transcription factors were consistent with the variation trends of CGA contents, suggesting their potential involvement in regulating CGA biosynthesis in *L. macranthoides*.

## 4. Discussion

### 4.1. Transcriptome and Metabolome Data Reveal Stage-Specific Molecular and Metabolic Variations in L. macranthoides Flowers

Clustering analysis of the expression patterns of DEGs in *L. macranthoides* showed that the WF and GF stages were further grouped into one cluster, which was clearly distinct from the FB stage. Among the three pairwise comparisons (WF vs. FB, GF vs. FB, and GF vs. WF), the variation in DAMs was the most significant in the GF vs. FB comparison. Previous research indicates that secondary metabolites, such as carotenoids and xanthophylls, exhibited relatively low concentrations at the FB stage, with a significant increase at the GF stage [33]. Additionally, apigenin derivatives and TMR flavonoids reached their highest levels at the GF stage [34]. These findings demonstrated a significant increase in multiple secondary metabolic components at the late floral developmental stage in *L. macranthoides*.

KEGG pathway enrichment analysis was performed separately on DEGs and DAMs from the transcriptome metabolome of *L. macranthoides*, and the results consistently pointed to the phenylpropanoid biosynthesis pathway. This pathway serves as a key hub in the plant secondary metabolic network, regulating the biosynthesis of key pharmacologically active components such as CGAs and flavonoids [35,36,37]. Notably, caffeoylquinic acid, the primary component of chlorogenic acid, reached its highest concentration at the WF stage, whereas ferulic acid, sinapic acid, cinnamic acid, and p-coumaric acid peaked at the GF stage. This stage-specific accumulation pattern is significant for medicinal applications: the GF stage may be optimal for harvesting multi-component activities, such as anti-inflammatory and antioxidant properties related to ferulic and sinapic acids, while the WF stage is more suitable for obtaining high concentrations of caffeoylquinic acid. This provides a scientific basis for the standardized cultivation of *L. macranthoides*.

### 4.2. Gene–Metabolite Coordination in the Phenylpropanoid Biosynthesis Pathway

This study focused on the phenylpropanoid biosynthesis pathway in *L. macranthoides* and identified 15 DAMs, including key precursor substances for CGA biosynthesis, as well as 11 DEGs encoding core enzymes involved in this pathway. The expression trends of four *PAL* gene family members (*PAL3–PAL6*) were fully consistent with the content trends of relevant metabolites in the pathway. The *PAL*, a core rate-limiting gene in CGA biosynthesis, has been functionally validated in multiple plant species. For instance, in cucumber (*Cucumis sativus*), the expression of the phenylpropanoid-biosynthesis-pathway-related gene *CsPAL1* was significantly positively correlated with the accumulation of p-coumaric acid, and its promoters contain multiple stress-responsive cis-acting elements [38]. Overexpression of *IbPAL1* could significantly increase the content of chlorogenic acid in *Ipomoea batatas* leaves [39]. The expression level of the *F5H* showed consistency with the content change trend of the downstream product, sinapic acid. This coordinated gene expression–metabolite accumulation pattern has also been validated in model plants. For instance, in the cadmium-tolerant *Zea mays* cultivar ZD958 under stress conditions, *F5H* expression was extremely significantly positively correlated with sinapic acid content [40]. In the *Arabidopsis Fah1* mutant, the loss of *F5H* function led to a stagnation in sinapate ester accumulation [41]. The expression of the *BnF5H1-3* in rapeseed directly regulates the biosynthesis of sinapic acid [42]. However, in this study, the expression levels of genes such as *C3H*, *4CL*, *COMT*, *CCR,* and *HQT* were inconsistent with the contents of related metabolites, which may be due to the cooperative expression patterns of genes, gene redundancy, and differences in metabolic flux distribution. Overexpressing the *HlMyb8* transcription factor gene in hop (*Humulus lupulus* L.) could activate the *4CL*, directing more metabolic flux toward flavonols and further affecting the direction of metabolic flux [43]. *CCR* and the other genes involved in the phenylpropanoid metabolic pathway exhibited coordinated expression, which collectively affected the biosynthesis and accumulation of metabolites such as lignin and flavonoids in grapes [44]. These results provide a new perspective for understanding the complexity of plant gene expression and the mechanisms underlying the accumulation of related metabolites.

### 4.3. Transcription Factors Regulate CGA Biosynthesis

WGCNA identified the “blue module”, which exhibited a correlation coefficient (r) > 0.89 with sinapic acid, p-coumaric acid, and cinnamic acid and contained 71 transcription factors belonging to 11 families. Members of the MYB family (seven genes) deserve special attention. Recent studies on *L. macranthoides* have shown that *LmMYB15* and *LmMYB111* exhibited a significant correlation with chlorogenic acid content, and their overexpression in tobacco could significantly increase the accumulation of chlorogenic acid [45,46]. Furthermore, for other transcription factor families in this module, such as bHLH, WRKY, and AP2/ERF, previous studies have also shown that these families were involved in regulating the CGAs. For instance, overexpression of *TabHLH1* in *Taraxacum antungense* Kitag significantly increased the contents of CGA and luteolin [47]. In tobacco, as a downstream target gene, MYB4 may form a complex with bHLH after being activated via *NtWRKY33a*, thereby coordinately regulating key enzymes in the CGA biosynthesis pathway (such as HCT) [48]. Additionally, both overexpression and knockout of the *NtERF13a* gene have demonstrated that *NtERF13a* enhances plant resistance to salt and drought stresses and promotes the biosynthesis of CGA, flavonoids, and lignin in tobacco [49]. In peach, PpbHLH14 interacts with PpMYB308 to form a heterodimer, which directly binds to the promoter of PpHCT5, thereby activating its expression [50]. In peanut, AhMYB10 directly interacts with AhbHLH35 to form a heterodimer, while AhWRKY40 enhances the DNA-binding capacity of the complex by binding to AhMYB10 within it. The combined action of these three factors increases the promoter activity of HCT and HQT by 4.2-fold [51]. These results indicate that a complex regulatory network exists for CGA biosynthesis in *L. macranthoides* at three different floral development stages, and that transcription factors such as MYB, bHLH, and WRKY may play crucial roles in this network.

## 5. Conclusions

This integrated transcriptomic and metabolomic study elucidated the dynamic regulatory network of phenylpropanoid biosynthesis during flower development in *L. macranthoides*. Our results revealed distinct, stage-specific accumulation patterns for key phenolic acids. Caffeoylquinic acid was predominant in white flowers (WFs), while ferulic acid, sinapic acid, cinnamic acid, and p-coumaric acid all reached their peak levels in golden flowers (GFs). These findings provide molecular evidence for determining optimal harvest times. Additionally, we demonstrated coordinated expression between structural genes, including *PAL* and *F5H*, and the accumulation patterns of their corresponding metabolites. Furthermore, weighted gene coexpression network analysis (WGCNA) identified crucial transcription factors from MYB, bHLH, and WRKY families, which collectively form an intricate regulatory network. This study unveils the implicated molecular mechanisms of GCA biosynthesis and provides critical insights and genetic resources for cultivating high medicinal-content varieties of *L. macranthoides*.

## Data Availability

Data will be made available upon request.

## Supplementary Materials

The following supporting information can be downloaded at: https://www.mdpi.com/article/10.3390/genes16111339/s1, Table S1: Transcriptome quality control; Table S2: Annotation of all unigenes; Table S3: Statistics on the number of differential metabolites; Table S4: Differentially expressed genes in the phenylpropanoid metabolic pathway; Table S5: Differentially accumulated metabolites in the phenylpropanoid metabolic pathway; Figure S1: Pie chart of NR annotations; Figure S2: Bar chart of GO classification; Figure S3: Chart of KOG classification.
