# Supplementary figures and images for: Integrated Transcriptomic and Metabolomic Analyses Reveal Key Genes Involved in Phenylpropanoid Metabolism in Lonicera macranthoides Flowers
Source: Genes (Basel). 2025 Nov 6;16(11):1339. doi: 10.3390/genes16111339 (PMC12651994; doi:10.3390/genes16111339)

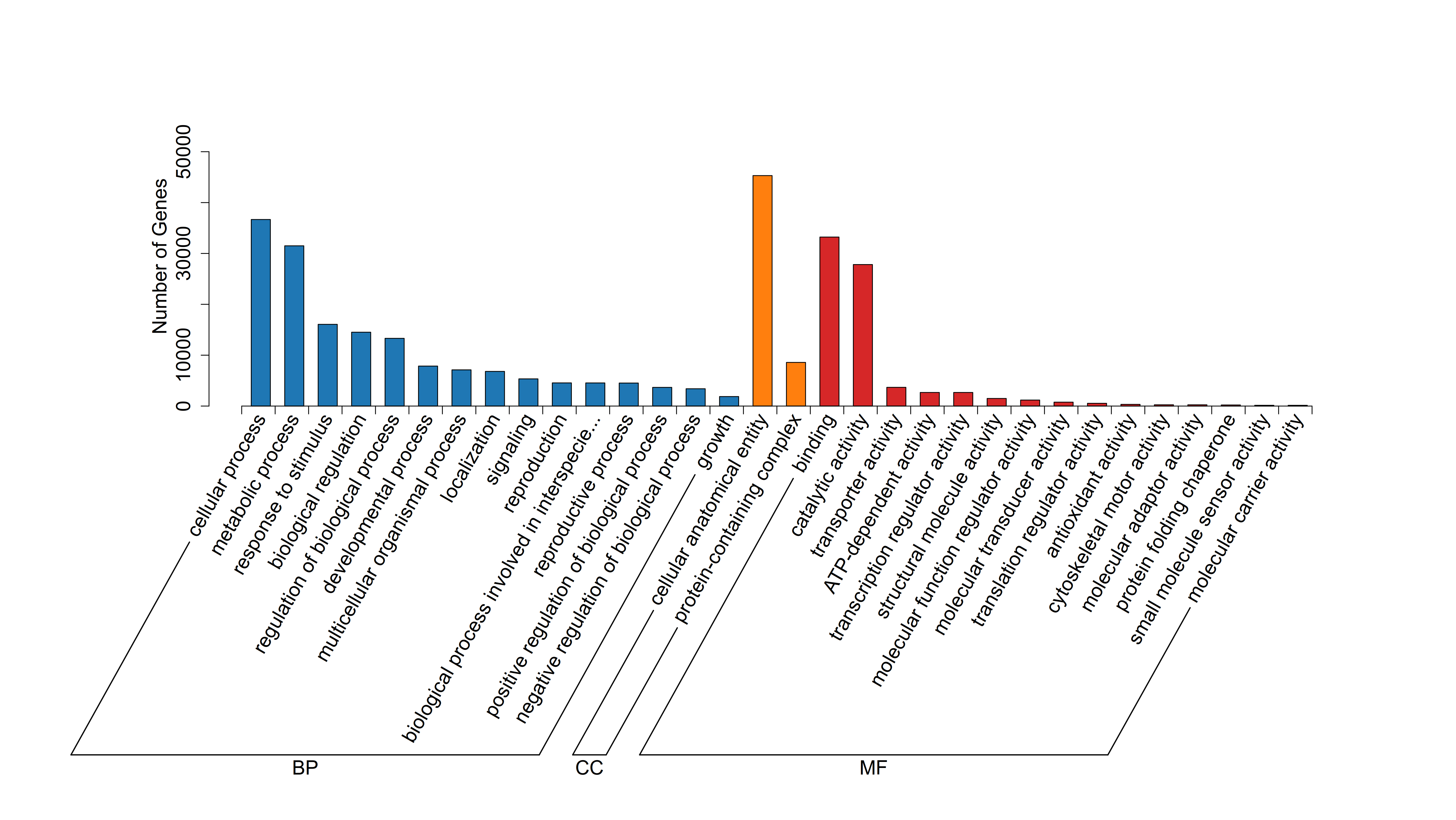

Supplement: Supplementary file 1 [file genes-16-01339-s001.zip › Figure S2.png]

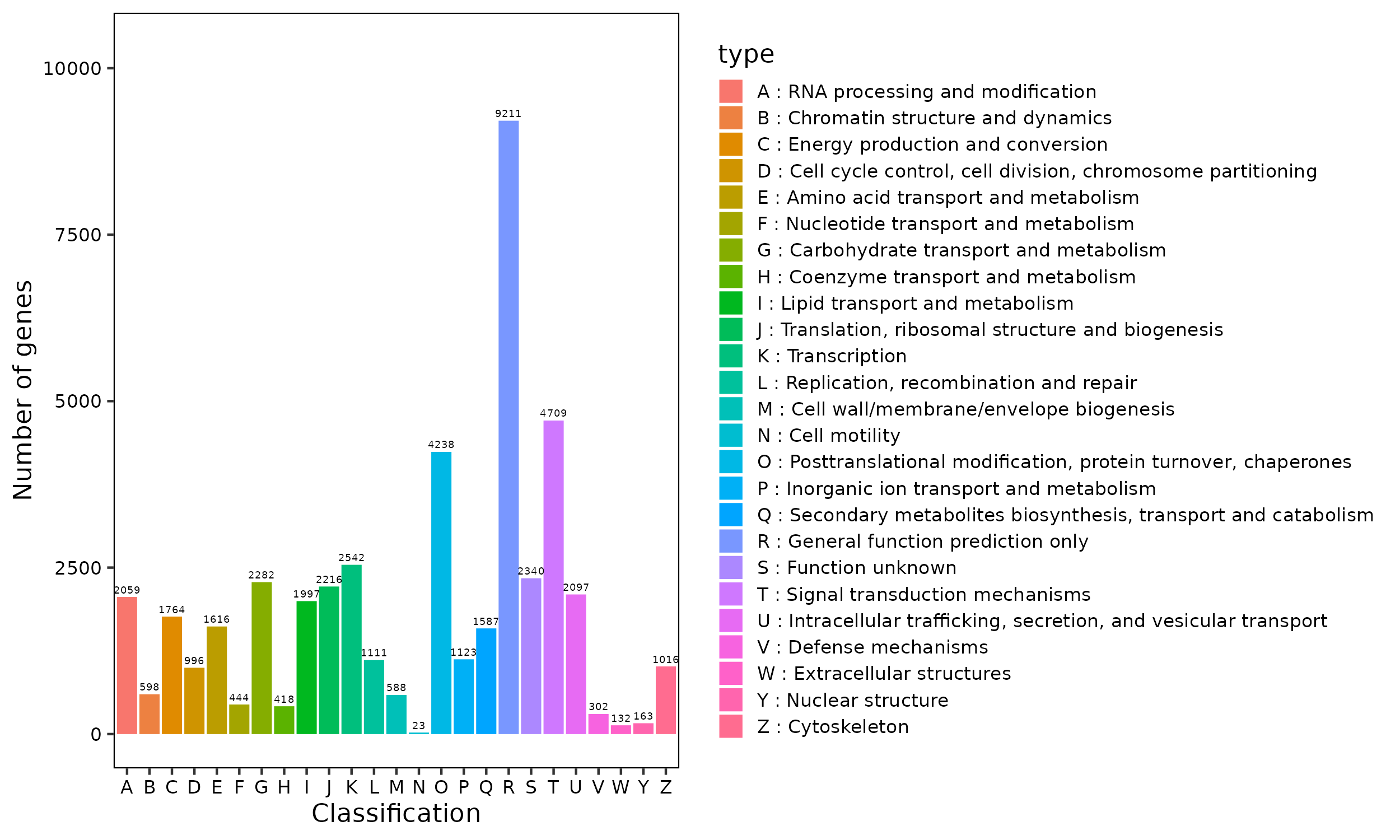

Supplement: Supplementary file 1 [file genes-16-01339-s001.zip › Figure S3.png]

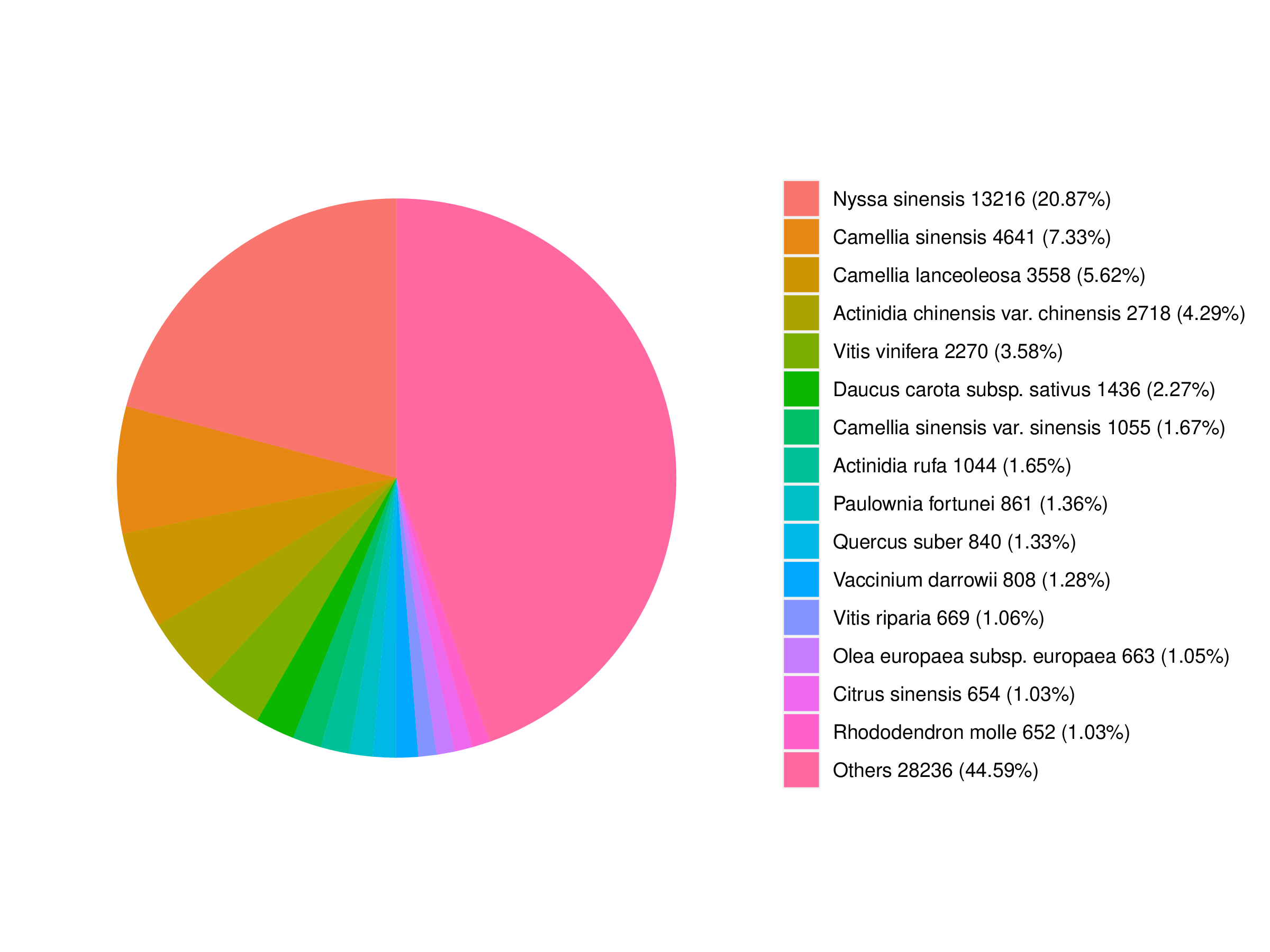

Supplement: Supplementary file 1 [file genes-16-01339-s001.zip › Figure S1.png]
